# Targeting eIF4F translation complex sensitizes B-ALL cells to tyrosine kinase inhibition

**DOI:** 10.1038/s41598-021-00950-y

**Published:** 2021-11-04

**Authors:** Thanh-Trang Vo, Lee-or Herzog, Roberta Buono, Jong-Hoon Scott Lee, Sharmila Mallya, Madeleine R. Duong, Joshua Thao, Moran Gotesman, David A. Fruman

**Affiliations:** Department of Molecular Biology and Biochemistry, University of California, Irvine, CA 92697 USA

**Keywords:** Haematological cancer, Cancer, Molecular medicine

## Abstract

The mechanistic target of rapamycin (mTOR) is a kinase whose activation is associated with poor prognosis in pre-B cell acute lymphoblastic leukemia (B-ALL). These and other findings have prompted diverse strategies for targeting mTOR signaling in B-ALL and other B-cell malignancies. In cellular models of Philadelphia Chromosome-positive (Ph+) B-ALL, mTOR kinase inhibitors (TOR-KIs) that inhibit both mTOR-complex-1 (mTORC1) and mTOR-complex-2 (mTORC2) enhance the cytotoxicity of tyrosine kinase inhibitors (TKIs) such as dasatinib. However, TOR-KIs have not shown substantial efficacy at tolerated doses in blood cancer clinical trials. Selective inhibition of mTORC1 or downstream effectors provides alternative strategies that may improve selectivity towards leukemia cells. Of particular interest is the eukaryotic initiation factor 4F (eIF4F) complex that mediates cap-dependent translation. Here we use novel chemical and genetic approaches to show that selective targeting of either mTORC1 kinase activity or components of the eIF4F complex sensitizes murine BCR-ABL-dependent pre-B leukemia cells to dasatinib. SBI-756, a small molecule inhibitor of eIF4F assembly, sensitizes human Ph+ and Ph-like B-ALL cells to dasatinib cytotoxicity without affecting survival of T lymphocytes or natural killer cells. These findings support the further evaluation of eIF4F-targeted molecules in combination therapies with TKIs in B-ALL and other blood cancers.

## Introduction

Many acute leukemias are driven by hyperactive tyrosine kinase (TK) signaling. Examples include Philadelphia Chromosome-positive B-cell acute lymphoblastic leukemia (Ph+ B-ALL), Ph-like B-ALL, and acute myeloid leukemia (AML) with Flt3 mutations^1–3^. Small molecule TK inhibitors (TKIs, e.g. imatinib, dasatinib, ruxolitinib, midostaurin) have clinical activity in certain TK-driven acute leukemias but are more effective in combination with other targeted or chemotherapeutic agents^4–11^. Identification of novel molecular targets and chemical agents to combine with TK inhibitors has potential to deepen clinical responses and might provide an alternative to toxic chemotherapies.

**Figure 1:**
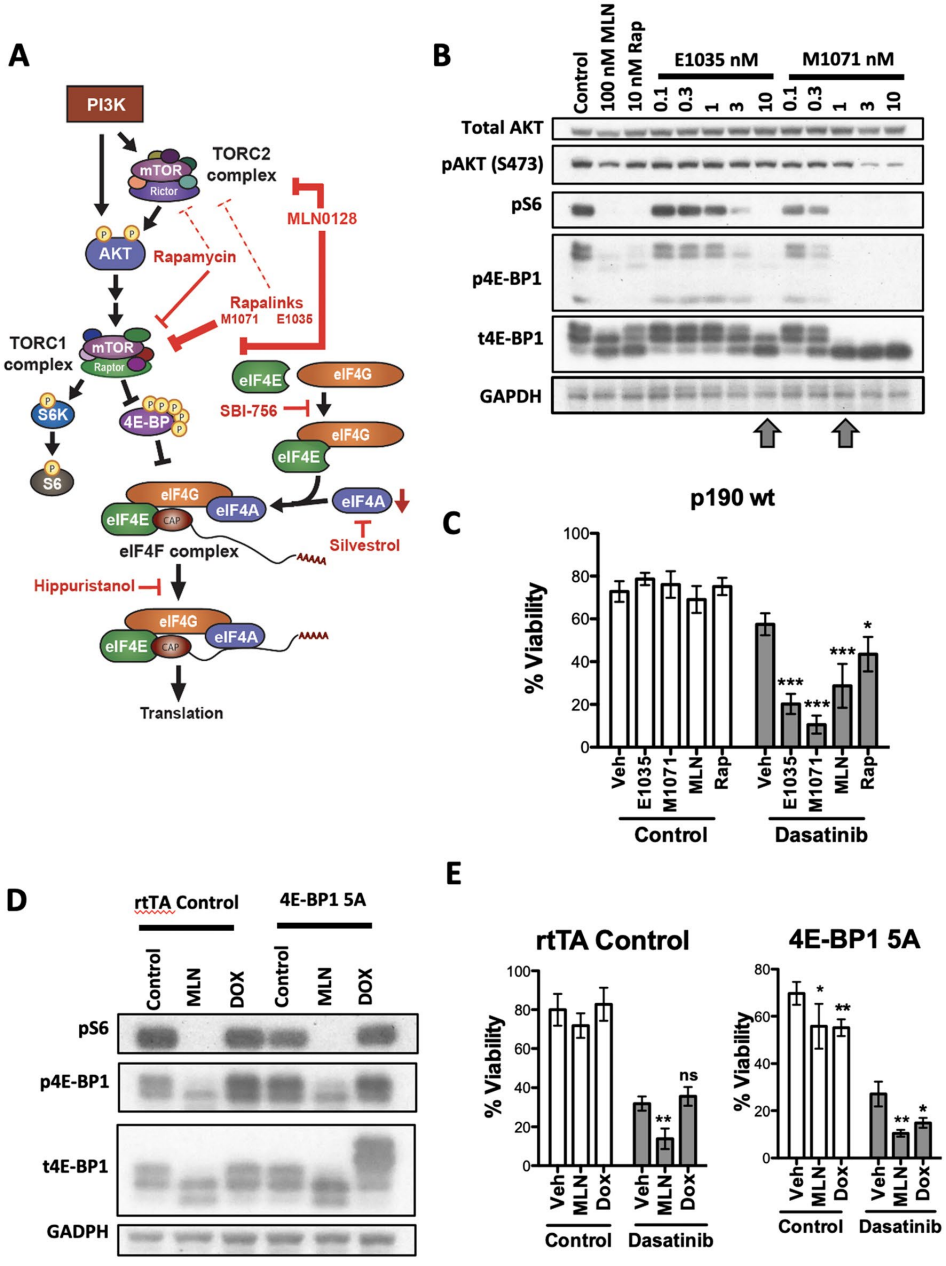
The cytotoxic effect of TOR-KI compound MLN0128 inhibition is fully recapitulated by selective mTORC1 kinase inhibitors (RapaLinks) or by genetic reactivation of 4E-BP1. (**A**) Schematic diagram of the PI3K/AKT/mTOR pathway, downstream mTORC1 effectors, and small molecule inhibitors used in this study. Notes: RapaLinks strongly inhibit mTORC1, and only inhibit mTORC2 at high concentrations; SBI-756 disrupts binding of eIF4G to eIF4E; silvestrol reduces the pool of free eIF4A by stabilizing its binding to mRNA; hippuristanol inhibits the helicase activity of eIF4A. (**B**) Western blot of p190 cells treated with TOR-KI (MLN0128, 100 nM), rapamycin (10 nM), or two RapaLink compounds (0.1–10 nM range) for 24 h. Blots were probed with antibodies to total and phosphoprotein targets shown at the left. Arrowheads indicate the RapaLink concentrations showing selective and complete inhibition of mTORC1 substrate phosphorylation. (**C**) Viability assay of p190 cells treated as indicated for 48 h. The percent viable cells was determined by Annexin-V/PI staining. The drug concentrations used were: dasatinib, 5 nM; E1035, 10 nM; M1071, 1 nM; MLN0128, 100 nM; rapamycin, 10 nM. (**D**) Western blot of p190 cells from single transgenic rtTA control and double transgenic (4E-BP1 5A) mice treated with control (DMSO, 0.1%) or MLN0128 (100 nM) or doxycycline (DOX, 1 µg/mL) for 24 h. Blots were probed with antibodies to total and phosphoprotein targets shown at the left. The 4E-BP1 5A expressed from the inducible transgenic is larger due to an epitope tag. (**E**) Viability assay of p190 cells treated as indicated for 48 h. Significance was calculated for C and E using two-way ANOVA (*p < 0.5, **p < 0.01, and ***p < 0.001, n = 3 per group).

The PI3K/AKT/mTOR signaling network is hyperactivated in leukemia and includes a number of kinases that are potential drug targets^12,13^. Phosphoinositide 3-kinases (PI3K) are a family of lipid kinases that promote reversible membrane recruitment of other signaling proteins including the serine/threonine kinase AKT (Fig. 1A)^14^. Inhibitors of the PI3Kδ isoform have entered clinical practice for mature B cell leukemias and lymphomas^15^, but are associated with significant toxicities^16^ and have not yet shown efficacy in acute leukemia. PI3Kδ inhibitors and AKT inhibitors have mainly been investigated in solid tumors where activating mutations of *PIK3CA* or *AKT1* are prevalent. The mechanistic target of rapamycin (mTOR) is a serine/threonine kinase that forms two distinct complexes, mTORC1 and mTORC2 (Fig. 1A)^17^. mTORC1 activity requires inputs both from nutrient sensing pathways and growth factor (or oncogene) signaling via PI3K/AKT. mTORC2 is partially dependent on PI3K activity and contributes to the phosphorylation and activation of AKT. Based on the diverse roles of mTOR complexes in cell proliferation, survival and metabolism, broad efforts have been made to develop and evaluate different classes of inhibitors.

Previous preclinical studies from our lab and others have shown that mTOR kinase inhibitors (TOR-KI) that target the active site of both mTORC1 and mTORC2 have greater anti-leukemic potential than rapamycin, an allosteric inhibitor that is a selective but partial inhibitor of mTORC1^13,18^. For example, the TOR-KI compound MLN0128 (later renamed TAK-228) enhances the efficacy of the TKI dasatinib in Ph+ and Ph-like B-ALL both in vitro and in mouse xenograft models, even in samples resistant to TKI treatment alone^10,19^. A likely mechanism for TKI-sensitization is that TOR-KI compounds suppress TK-independent survival signals provided by growth factors and nutrients in the leukemia niche. However, clinical trials of TOR-KI agents including TAK-228 (sapanisertib) and AZD2014 (vistusertib) have not demonstrated clear efficacy at tolerated doses^20–25^. It is not clear to what extent the efficacy and dose-limiting toxicities of TOR-KI agents are due to inhibition of mTORC1 or mTORC2.

These questions emphasize the importance of defining the role of individual mTOR complexes in sensitization to TKIs, and also to search for additional downstream targets that might offer an alternative route to selective anti-leukemic activity. In this study we focused on the mTORC1 signaling node and an important downstream effector, eukaryotic initiation factor 4F (eIF4F). This complex of proteins includes the cap-binding protein eIF4E, the scaffolding protein eIF4G, and the RNA helicase eIF4A. Numerous studies have demonstrated that eIF4F activity promotes leukemogenesis and drives cap-dependent translation of mRNAs involved in cell proliferation and survival^26–30^. Furthermore, efforts are underway to develop small molecule inhibitors of eIF4F components^31,32^. Here we use novel genetic and chemical tools to establish that targeting eIF4F has similar anti-leukemic effects compared to TOR-KI treatment, with great potential as an alternative approach to enhancing TKI efficacy.

## Results

### Selective mTORC1 inhibition phenocopies dual mTORC1/mTORC2 inhibition

Previously we demonstrated that TOR-KI compounds (PP242, MLN0128; Fig. 1A) sensitize Ph+ B-ALL cells to TKIs including imatinib and dasatinib^19,33^. Since TOR-KIs inhibit the kinase activity of both mTORC1 and mTORC2, we sought to determine whether TKI sensitization required inhibition of one or both targets. To compare different mTOR inhibitors alone or in combination with dasatinib, we used the p190 cell system in which primary murine leukemia cells are generated from bone marrow using a retroviral vector expressing p190-BCR-ABL. We treated p190 cells with mTOR inhibitors for 24 h prior to measurement of mTOR signaling readouts. We compared the TOR-KI compound MLN0128 with rapamycin, a natural product that is a potent but partial inhibitor of mTORC1, inhibiting phosphorylation of some substrates (such as S6 kinases) more than others (such as 4E-BP1)^34–36^. As expected, rapamycin strongly suppressed pS6 (S240/244), a substrate of S6 kinases. Rapamycin also suppressed p4E-BP1 (T37/46), though some degree of phosphorylation remained as assessed using a total 4E-BP1 antibody that detects distinct mobilities of 4E-BP1 phospho-species (Fig. 1B). Although rapamycin can inhibit mTORC2 under some conditions, it had no effect on the mTORC2 substrate AKT-S473 in p190 cells (Fig. 1B). The TOR-KI compound MLN0128 (100 nM) also blocked pS6, and reduced p4E-BP1 to a greater extent than rapamycin (which is most apparent using the total 4E-BP1 antibody). MLN0128 partly reduced pAKT (Fig. 1B). To assess the effect of selective mTORC1 kinase inhibition we tested two RapaLink compounds E1035 and M1071. Both RapaLinks selectively suppressed phosphorylation of mTORC1 readouts (p-4E-BP1, pS6) at concentrations that did not affect mTORC2 substrate phosphorylation (pAKT-S473). E1035 was mTORC1-selective at 3 and 10 nM, and M1071 starting at 1 nM (Fig. 1B, arrowheads). Both Rapalinks, especially M1071, caused more complete dephosphorylation of p4E-BP1 compared to the effect of MLN0128, as judged by mobility shift of the total 4E-BP1 protein signal (Fig. 1B).

Next we tested the effects of single and dual compound treatments on p190 cell viability after 48 h. None of the mTOR inhibitors affected viability as single agents, but all enhanced cytotoxicity by dasatinib (Fig. 1C). The two Rapalink compounds had concentration-dependent activity in this assay (Fig. S1A,B) and were equally effective as MLN0128 (Fig. 1C). These data indicate that selective mTORC1 inhibition is sufficient to recapitulate the cell death sensitization observed with dual mTORC1/mTORC2 inhibitors.

### Genetic inhibition of eIF4E function sensitizes to dasatinib

mTORC1 has numerous substrates that control various aspects of cell proliferation and survival^13,17^. The 4E-BPs are key mTORC1 substrates whose phosphorylation releases eIF4E to form the eIF4F complex that also contains eIF4G1 and eIF4A. We first assessed the role of the 4E-BP/eIF4E axis using an inducible system to express a constitutively active mutant of 4E-BP1 in which five serine or threonine phosphorylation sites are changed to alanine (“5A” mutant). We generated p190 leukemia cell pools from double transgenic mice that ubiquitously express the reverse transactivator (“tet-on”) protein rtTA (Rosa26-rtTA) along with a tetracycline response element (TRE)-linked cDNA encoding 4E-BP1-5A^37^. Control cells were generated from Rosa26-rtTA single transgenic littermates. Western blots showed robust expression of the 4E-BP1-5A protein after doxycycline (DOX) treatment in the double transgenic cells (Fig. 1D). We then tested expression and function of the 4E-BP1 mutant in a cap-binding assay. Treatment of control cells with MLN0128 but not DOX displaced eIF4G1 from eIF4E (Fig. S1C). In the double transgenic cells, DOX induced expression of the 4E-BP1 mutant displaced eIF4G1, to a similar or greater extent than MLN0128 treatment. This biochemical effect correlated with cytotoxicity. DOX treatment of double transgenic p190 cells phenocopied the effect of MLN0128 on cell viability, both alone and in combination with dasatinib (Fig. 1E). DOX treatment had no effect on viability in control p190 cells. These results are consistent with the model that 4E-BP1 activation is a critical pro-death mechanism of mTORC1 inhibition. Of note, a selective inhibitor of S6K1 (LY2584702)^38^ did not reduce p190 cell viability when added alone or together with dasatinib (Fig. S1D, E).

**Figure 2:**
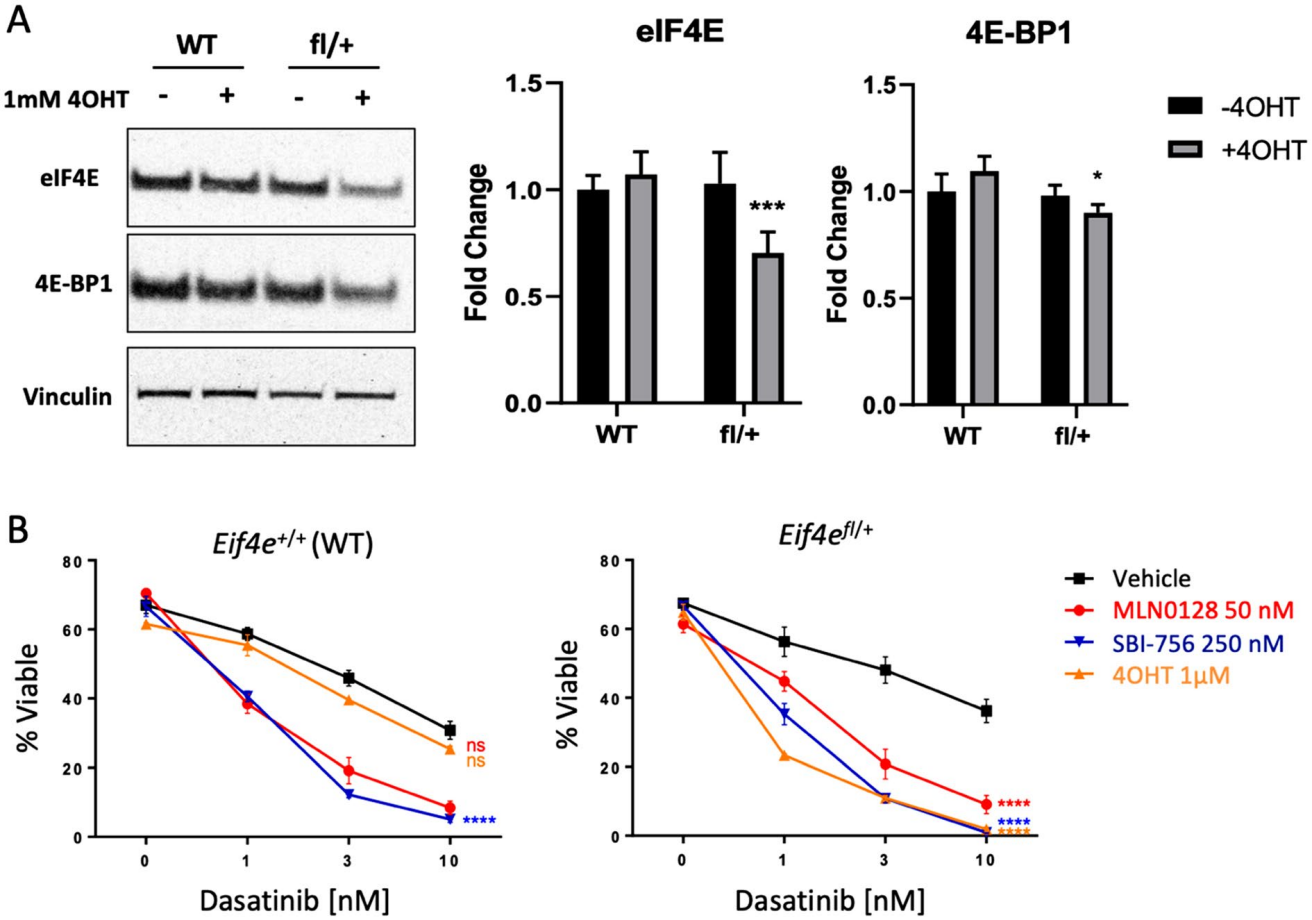
Reduced *Eif4e* gene dosage sensitizes BCR-ABL-dependent cells to dasatinib. (**A**) eIF4E expression was assessed by Western blot in pools of p190 cells from Rosa26-CreER mice with WT (+/+) or heterozygous floxed (fl/+) *Eif4e*. Cells were treated without or with 4OHT for 72 h prior to lysis. Graph on the right shows relative expression of eIF4E and 4E-BP1 over several independent p190 pools (n = 6–9). Fold change calculated using total Erk or vinculin as loading control. Data are expressed as mean ± S.E.M. Significance was calculated using paired t-test (*p < 0.05; ***p < 0.001, n = 6–9) (**B**) Viability assay of p190 cells treated as indicated for 48 h. Significance was calculated using two-way ANOVA (****p < 0.0001, n = 4 for each graph).

As a distinct approach to interfere with eIF4E function, we used a genetic eIF4E loss of function model. We obtained mice with a conditional floxed allele of *Eif4e* from the NorCOMM2 mouse repository (C57BL/6 N-*Eif4e*^tm1c^^(EUCOMM)Hmgu^/Tcp) and crossed them with another knockin strain in which inducible Cre is expressed in all tissues (Rosa26-CreERT2)^39^. We generated p190 cells from Cre-positive *Eif4e*^fl/+^ and control *Eif4e*^+/+^ mice and assessed deletion efficiency by Western blot. After treatment with 4OHT (1 µM) for 72 h eIF4E expression was reduced by 30–40% in *Eif4e*^fl/+^ cells but unaltered in control p190 cells (Fig. 2A). Notably, deletion of one allele of *Eif4e* sensitized p190 cells to dasatinib cytotoxicity, and the magnitude of this effect was similar to that achieved in cells treated with MLN0128 (Fig. 2B). Consistently, 4OHT treatment sensitized *Eif4e*^fl/+^ cells but not *Eif4e*^+/+^ p190 cells to dasatinib (Fig. 2B; Supplementary Figure 2). Thus, reduced *Eif4e* gene dosage strongly sensitizes cells to BCR-ABL inhibition.

### Chemical inhibition of eIF4F components sensitizes to dasatinib

**Figure 3:**
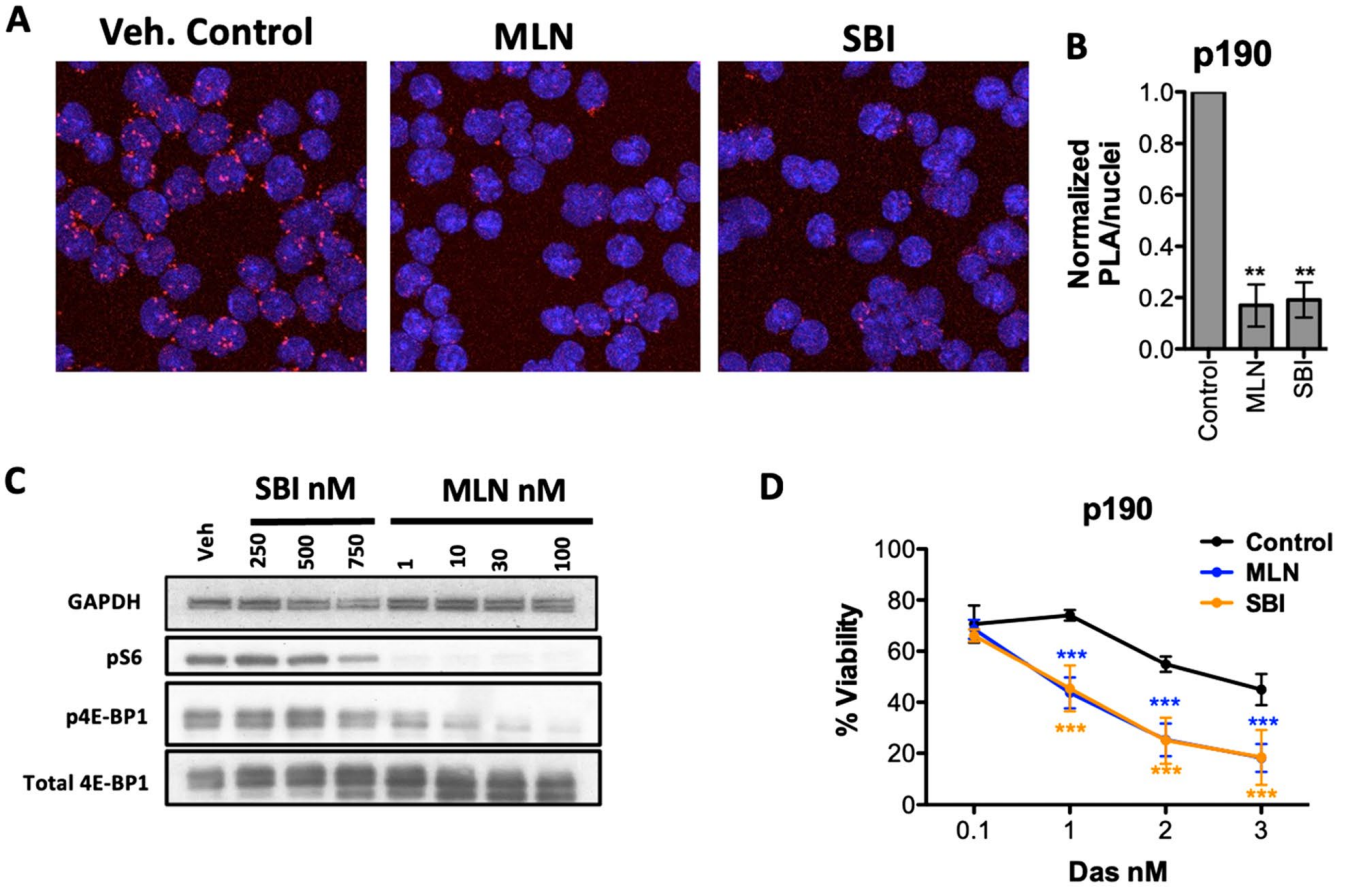
SBI-756 disrupts eIF4F and sensitizes to dasatinib without inhibiting mTORC1. (**A**) Proximity Ligation Assay (PLA) was done using p190 cells treated as indicated for 4 h and analyzed by confocal microscopy. Red dots indicate co-localization of eIF4E and eIF4G in situ. Blue = nuclei stained with DAPI. (**B**) Quantitation of PLA data normalized to the number of nuclei (5 fields, 3 replicates). A one-sample *t test* was done to show that treatment is significantly different from normalized control. **p < 0.01, n = 3. (**C**) Western blot of p190 cells treated with SBI-756 or MLN0128 at the indicated concentrations for 4 h. (**D**) Viability assay of p190 cells treated as indicated for 48 h. Significance was calculated using two-way ANOVA (***p < 0.001, n = 3). (**A**, **B**, **D**) MLN0128 was used at 100 nM, and SBI-756 at 500 nM.

The genetic approaches described above build on previous evidence that the eIF4F complex has important functions in BCR-ABL-mediated transformation^40–43^. In accord, we found that two natural products that inactivate eIF4A helicase, silvestrol and hippuristanol, sensitized p190 cells and human Ph+ and Ph-like B-ALL cell lines to dasatinib (Supplementary Figure 3). As an alternative chemical approach, we tested the compound SBI-756 that binds eIF4G1 and prevents its interaction with eIF4E^29,44^. To confirm the on-target effects of SBI-756 in p190 cells, we compared the impact of SBI-756 and MLN0128 on eIF4F assembly and mTORC1 substrate phosphorylation. Using proximity ligation assay (PLA), we found that both SBI-756 and MLN0128 significantly blocked the association of eIF4E with eIF4G1 in intact p190 cells (Fig. 3A,B). In contrast, SBI-756 did not suppress MLN0128-sensitive phosphorylation of S6 and 4E-BP1 (Fig. 3C). Thus, at concentrations that do not impact mTORC1 activity, SBI-756 has similar potential as a TOR-KI to target the eIF4F complex.

### SBI-756 suppresses leukemia outgrowth and sensitizes mouse and human B-ALL cells to dasatinib

**Figure 4:**
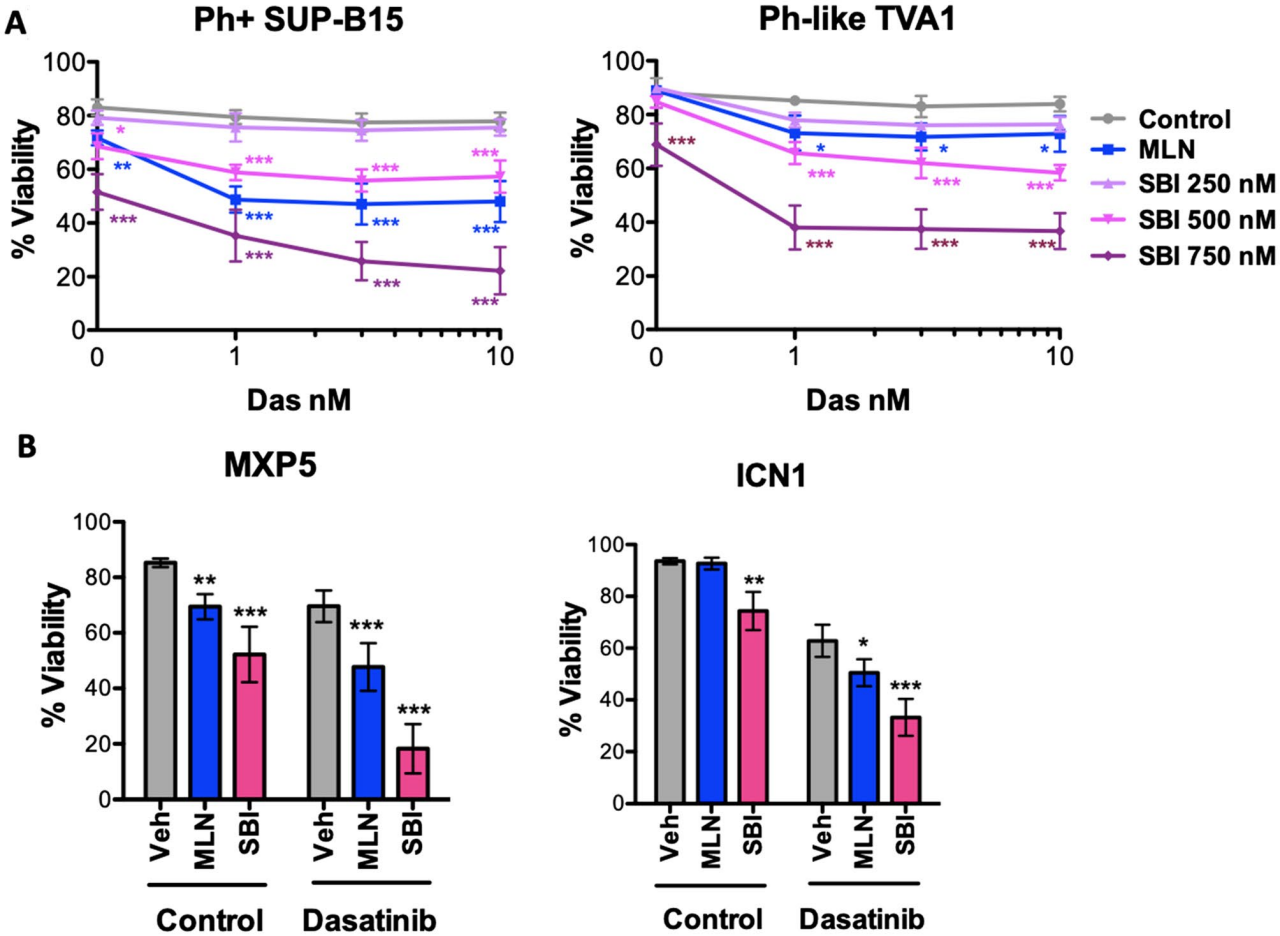
SBI-756 sensitizes to dasatinib in human Ph+ and Ph-like B-ALL cell lines, and stromal-dependent Ph+ B-ALL cells. (**A**) Viability assay of SUP-B15 and TVA1 cells after 48 h of treatment as indicated. (*p < 0.5, **p < 0.01, and ***p < 0.001, n = 3–4) (**B**) Viability assay of MXP5 and ICN1 cells cultured on OP9 stromal cells. Drug concentrations: dasatinib, 10 nM (except 1 nM for MXP5); MLN0128, 100 nM; SBI-756, 750 nM. Significance was calculated for A and B using two-way ANOVA (*p < 0.5, **p < 0.01, and ***p < 0.001, n = 3–5).

In colony formation assays, SBI-756 potently blocked leukemic transformation of bone marrow cells by BCR-ABL, similar to the effects of dasatinib, MLN0128 or Rapalinks (Fig. S4). In viability assays of established p190 cells, treatment with SBI-756 (250 nM) sensitized to dasatinib to a comparable extent as MLN0128 or deletion of one allele of *Eif4e* (Figs. 2B, 3D; Fig. S2). To assess SBI-756 potential in human leukemia cells, we first carried out viability assays using the Ph+ B-ALL cell line SUP-B15 and the Ph-like B-ALL cell line TVA1^10^. Both cell lines were resistant to dasatinib alone at concentrations up to 10 nM (Fig. 4A). MLN0128 (100 nM) had a modest sensitization effect on both cell lines, which was recapitulated by SBI-756 (500 nM in SUP-B15 cells; 250 nM in TVA-1 cells; Fig. 4A). Next, we compared single and combination treatments in two patient-derived, stroma-dependent Ph+ B-ALL cell lines (MXP5, ICN1). In both lines, SBI-756 alone had a significant cytotoxic effect and this was greater than the effect of MLN0128 in ICN1 cells (Fig. 4B). In both cell lines, the combination of SBI-756 was more cytotoxic compared to dasatinib alone or to dasatinib combined with MLN0128 (Fig. 4B).

### Inhibitors of the mTORC1/eIF4F axis are selectively toxic to B lineage cells

**Figure 5:**
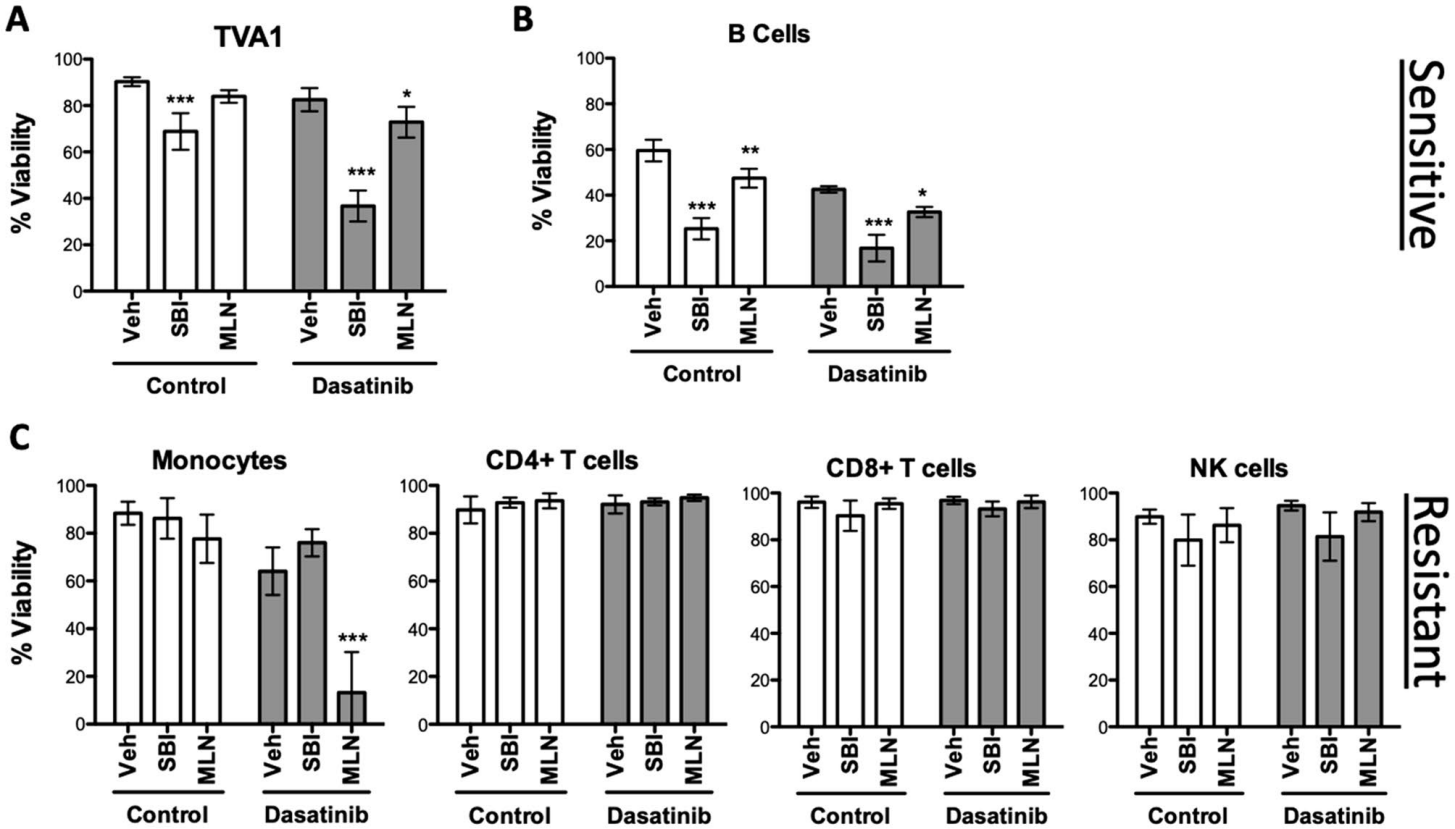
SBI-756 is selectively toxic to B-lineage cells. (**A**) Viability of assay of TVA1 Ph-like B-ALL cell line as a positive control for SBI-756 cytotoxicity. (**B**, **C**) PBMCs from healthy donors were cultured for 48 h as indicated. Viability of leukocyte subsets was determined by multiparameter flow cytometry. Drug concentrations: dasatinib, 10 nM; MLN0128, 100 nM; SBI-756, 750 nM. Significance was calculated using two-way ANOVA (*p < 0.5, **p < 0.01, and ***p < 0.001, n = 3).

To assess the selectivity of pathway inhibitors for leukemic cells, we carried out viability assays using PBMCs derived from healthy donors. TVA1 cells were used as a positive control for SBI-756 sensitivity (Fig. 5A). After 48 h treatment, MLN0128 and SBI-756 both reduced survival of B cells and increased killing by dasatinib (Fig. 5B). However, these inhibitors had little or no effect on CD4 T cells, CD8 T cells, NK cells or monocytes, with the exception that MLN0128 combined with dasatinib was cytotoxic to monocytes. Together with the cytotoxicity experiments using murine and human B-ALL cells, and our previous studies of human B-cell lymphomas^29^, these data suggest that cells of the B lymphocyte lineage are selectively sensitive to SBI-756 cytotoxicity.

## Discussion

In this study we have provided evidence that selective inhibition of either mTORC1 or eIF4F achieves comparable anti-leukemic activity as dual mTORC1/mTORC2 inhibitors (TOR-KI) in cellular models of Ph+ and Ph-like B-ALL. RapaLink molecules with ~ tenfold selectivity for mTORC1 versus mTORC2 suppress BCR-ABL-driven colony formation at low nM concentrations and sensitize p190 cells to killing by dasatinib. In two distinct genetic models of eIF4F inhibition (inducible expression of 4E-BP1 mutant; conditional deletion of one allele of *Eif4e*), reduced eIF4F function sensitizes p190 cells to dasatinib. Similarly, the eIF4G-binding compound SBI-756 disrupts formation of eIF4F and promotes dasatinib-mediated killing of mouse p190 cells and Ph+ or Ph-like B-ALL cells. Together these findings support the conclusion that TKI sensitization by TOR-KI is primarily due to inhibition of the mTORC1/eIF4F axis. Furthermore, the cellular activity of SBI-756 provides proof of concept for further development of eIF4F-targeted agents in TK-driven B-ALL.

Treatment of B-ALL cells with SBI-756 in the 250–500 nM range strongly reduced formation of the eIF4F complex in cells without impacting mTORC1 signaling outputs (pS6, p4E-BP1). These findings are in accord with previous studies of SBI-756 in melanoma^44^ and B cell lymphoma^29^ cell lines. The selective inhibition of one mTORC1 effector pathway might provide advantages by reducing toxicities associated with mTORC1 inhibition. Similar to TOR-KI compounds, SBI-756 seems to have more general cytotoxic activity towards B lineage cells, not only leukemia and lymphoma cells but also normal human B cells in PBMC cultures (Fig. 5,^29^). SBI-756 did not affect the survival of T cells, NK cells or monocytes (Fig. 5,^29^). MLN0128 had no effect on T cells or NK cells but did cause monocyte cell death in combination with dasatinib. Although SBI-756 is a helpful tool compound, molecules with better pharmacokinetic properties in mice are needed to further evaluate the potential of eIF4G1 as a target in vivo, and toxicities relative to mTOR inhibitors.

Other components of the eIF4F complex are promising targets for oncology drug development. One of these is the RNA helicase eIF4A^45^. Two natural products that bind eIF4A, hippuristanol and silvestrol, enhanced dasatinib killing of p190 cells (Fig. S3A). Synthetic rocaglate compounds that target eIF4A have received considerable attention as cancer therapies, and a novel inhibitor eFT226 is in clinical development (NCT04092673)^45–47^. A number of compounds have been reported to bind eIF4E but most have had low potency and/or poor cell penetrance^48,49^. Recently, novel eIF4E antagonists have been disclosed and are in preclinical development. It will be interesting to test whether these eIF4E inhibitors potentiate TKI activity in B-ALL and other cancers.

## Methods

### Mice

Mice were housed and studied in compliance with a protocol approved by the Institutional Animal Care and Use Committee of the University of California, Irvine. All methods were carried out in accordance with relevant guidelines and regulations, and in accordance with ARRIVE guidelines (https://arriveguidelines.org). For inducible expression of mutant 4E-BP1, we used double transgenic mice (Rosa26-rtTA, TRE-4EBP1M) as described^37^, with littermate single transgenic (Rosa26-rtTA) as the control. We also generated a novel strain of *Eif4e* conditional knockout (cKO) mice**.** We obtained cryopreserved sperm from mice with a conditional-ready tm1c allele of *Eif4e,* with exons 5 and 6 floxed, from the NorCOMM center in Toronto (affiliated with the Knockout Mouse Consortium). See link: http://www.cmmr.ca/gene-detail.php?gene=MGI:95305. We reanimated this line by in vitro fertilization of wild-type C57Bl/6N oocytes and performed breedings with Rosa26-CreER mice (Jackson Labs) to obtain an allelic series with different gene dosage (+/+, fl/+, fl/fl).

### Chemicals

We obtained rapamycin, dasatinib and MLN0128 from LC Laboratories (Woburn, MA, USA).

SBI-0640756 was provided by Dr. Ze’ev Ronai (Sanford Burnham Prebys Institute) or purchased from Selleck with comparable results. Dimethyl sulfoxide (DMSO) was obtained from Fisher Scientific (Waltham, MA, USA). The rapalinks compounds E1035 and M1071 (also known as RapaLink-1) were kind gifts from Dr. Kevan Shokat from the University of San Francisco, CA. Hippuristanol and Silvestrol were kindly provided by Jerry Pelletier (McGill University).

### Cell culture

Mouse bone marrow (BM) was obtained by flushing cells from the long bones (tibias and femurs) of 3- to 4-wk-old mice. The Balb/c strain was used to generate wild-type p190 pre-B leukemia cells. Genetically modified strains were used to generate p190 cells with inducible expression of mutant 4E-BP1 or inducible deletion of eIF4E. Cells were spinoculated with retroviral supernatant (using the vector MSCV-p190BCRABL-IRES-hCD4) in the presence of 5 μg/ml polybrene for 45 min. at 450 × g and 37 °C with RPMI20 culture media supplemented with recombinant mouse IL-7 (10 ng/ml, R&D Systems) to promote cell cycle entry. After incubation overnight, cells were spun down and grown in RPMI20 media with 10 ng/mL IL-7 for 1 week. They were then grown without IL-7 for an additional week with gradual reduction of serum to 10% FBS. The surviving cells were analyzed by flow cytometry to verify uniform human CD4 expression. Human Ph+ B-ALL cell lines SUP-B15 and BV173 were purchased from cell line repositories (ATCC and DSMZ, respectively). We established the Ph-like B-ALL cell line TVA1 from a PDX model as described previously^10^. SUP-B15 and TVA1 cells were cultured in RPMI-1640 (Corning) supplemented with 10% heat-inactivated FBS, 10 mM HEPES, 2 mM L-glutamine and 100 I.U./ml penicillin/streptomycin. MXP5 and ICN1 are patient-derived Ph^+^ ALL cells cultured on OP9 stroma as we have described previously^50^. All cell lines were grown in a humidified 37 °C incubator with 5% CO2. Cells were routinely tested to ensure absence of mycoplasma and validated by STR profiling, and were maintained at or below 2 × 10e6 cells/ml.

### Colony forming assay

BCR-ABL infected mouse bone marrow cells were resuspended in primary media at 5 × 10^4^ cells/condition with recombinant human IL-7 at 10 ng/mL (Thermo Fisher Scientific). Cells and inhibitors at the designated concentrations were then suspended in methylcellulose Methocult M3630 (StemCell Technologies) and vortexed for 90 s prior to plating in duplicate in 24-well plates. Colonies were counted around 9 days after plating and number of colonies was averaged for counts from two independent sets of observations.

### Immunoblotting

Cells were lysed in radio-immunoprecipitation assay buffer (150 mM NaCl, 1.0% IGEPAL® CA- 630, 0.5% sodium deoxycholate, 0.1% SDS, and 50 mM Tris, pH 8.0, 2 mM EDTA, 50 mM NaF) supplemented with protease inhibitor cocktail (Calbiochem, USA) and phosphatase inhibitor cocktails 2 and 3 (Sigma-Aldrich). Protein concentrations were normalized using a Bradford protein assay (Bio-Rad, Hercules, CA). Lysates were prepared at 1 µg/µl concentration in 1X XT Sample Buffer (Bio-Rad) and 5% BME (Sigma-Aldrich). Lysates were run on 8–12% Bis–Tris gels and transferred onto nitrocellulose membranes (Bio-Rad). Antibodies to the following phosphoproteins and total proteins were used: phospho-Akt (S473), Akt, phospho-rS6 (S240/244), phospho-4E-BP1 (Thr 37/46), 4E-BP1, GAPDH, Actin, eIF4E, eIF4G1, ERK1/2, and vinculin (Cell Signaling Technology, Beverly, MA, USA). We used the anti-mouse IgG and anti-rabbit IgG secondary HRP-conjugated antibodies from Promega (Madison, WI, USA). Antibody dilutions were performed according to the manufacturer’s instructions. Immunoreactive bands were visualized using Amersham ECL Prime Western Blotting Detection Reagent (GE Healthcare Life Sciences) or Super Signal West Femto Maximum Sensitivity Substrate (Thermo Fisher Scientific) and detected using a Nikon D700 SLR camera as described previously^51^. Images were processed and densitometry was quantified using ImageJ software (NIH).

### Cell viability

Cell viability assays were performed in 96-well U-bottom plates, with 6 × 10e4 cells in 200 µl. Cells were harvested by centrifuging the 96-well plate in a plate spinner centrifuge at 1700 rpm for 5 min. Cells were washed and stained with Annexin V conjugated to Alexa Fluor™ 647 nm (Thermo Fisher Scientific) and 0.1 mg/ml propidium iodide (PI)-staining solution (Life Technologies). Cells were then analyzed on a FACSCalibur flow cytometer (Becton–Dickinson, San Jose, CA) or ACEA NovoCyte Flow Cytometer (ACEA Biosciences, San Diego, CA). FlowJo software v.5.7.2 (TreeStar, Ashland, OR) was used to process the data. Percentage of viable cells was determined based on the fraction of total cells that were Annexin V-negative and PI-negative.

### Duolink proximity ligation assay (PLA)

2 × 10e6 cells were treated for four hours with the treatments indicated. Following treatment, cells were washed with Phosphate Buffered Saline 1x (PBS) (Corning) and fixed with 4% paraformaldehyde (Thermo Fisher Scientific). Comet Slides (2 Well) (Trevigen, Gaithersburg, MD) were pre-coated with Poly-L-Lysine 0.1% solution (Sigma Aldrich), and fixed cells were allowed to adhere for at least 20 min. Duolink PLA protocol was followed, as described previously^52^. Briefly: Cells were blocked using Duolink blocking solution, followed by probing with primary antibodies for the proteins of interest from two different origins (for eIF4G1, Cell signaling Technologies Cat. #2858, 1:200 dilution; for eIF4E, BD Biosciences Cat. #610269, 2.5 µg/ml final). Cells were next incubated with Duolink In Situ PLA Probe Anti-Rabbit PLUS Affinity purified Donkey anti-Rabbit IgG (H + L) (Cat. # DUO92002) and Duolink In Situ PLA Probe Anti-Mouse MINUS Affinity purified Donkey anti-Mouse IgG (H + L) (Cat. # DUO92004) and allowed to ligate using ligation mix (1:5 dilution). Next, amplification and washes were performed as instructed by manufacturer and the slides were mounted using media containing DAPI (Sigma Aldrich, DUO82040-5ML). Slides were imaged using Leica TCS SP8 confocal microscope. Signal obtained was quantified using ImageJ software, and normalized to the number of cells per field (using DAPI nuclei staining). Images shown indicate the signal (Orange Duolink™) and nuclei for each field imaged, while graphs presented indicate ratio values of signal per cell in each field imaged.

### Statistical analysis

The number “n” of biological replicates for each experiment is indicated in the figure legends. Two-way ANOVA for multiple comparisons was performed where indicated while considering sample independence, variance equality and normality. Student *t*-tests were applied to population means assuming equal variance (standard deviations within twofold). The use of one- versus two-sample tests, and paired versus unpaired comparisons, was justified by the experimental design.

## Supplementary Information

Supplementary Information.
