# Supplementary material for: Targeting eIF4F translation complex sensitizes B-ALL cells to tyrosine kinase inhibition
Source: Sci Rep. 2021 Nov 4;11:21689. doi: 10.1038/s41598-021-00950-y (PMC8569117; doi:10.1038/s41598-021-00950-y)
Supplement: Supplementary file 1 — Supplementary Information. [file 41598_2021_950_MOESM1_ESM.pdf]

Supplemental Information

Supplementary Figure Legends

Figure S1: The 4E-BP signaling axis mediates cytotoxicity of mTORC1 inhibition. (A, B) Viability assay of p190 cells treated with control (DMSO) or dasatinib (5 nM) and titrated amounts of RapaLink compounds E1035 and M1071. (C) Cap-binding assay in lysates of p190 cells from single transgenic (Rosa) and double transgenic (4E-BP1 5A) mice treated with control (DMSO, 0.1%) or MLN0128 (100 nM) or doxycycline (DOX, 1 µg/mL) for 24 hours. Blots were prepared from the cap-binding fraction and the total lysate, and probed with antibodies to eIF4G and eIF4E. (D) Western blot showing that LY2584702 inhibits S6K activity as measured by phosphorylation of ribosomal S6 protein (S240/244), but does not inhibit 4E-BP1 phosphorylation. (E) Viability assay of p190 cells treated with vehicle (DMSO) or dasatinib (5 nM) and the indicated inhibitors of mTOR or S6K. Significance was calculated for A, B and E using two-way ANOVA (* $p < 0.5$, ** $p < 0.01$, and *** $p < 0.001$. $n = 3$).

Figure S2: Reduced *Eif4e* gene dosage or treatment with SBI-756 sensitize BCR-ABL-dependent cells to dasatinib. Viability assays were performed using additional pools of WT and fl/+ p190 cells as in Figure 2. In these experiments, SBI-756 (250 nM) was also tested as a single agent and in combination with dasatinib. Significance was calculated using two-way ANOVA (* $p < 0.05$; *** $p < 0.001$ **** $p < 0.0001$, $n = 3 – 6$ for each).

Figure S3: Inhibitors of eIF4A helicase function sensitize mouse and human B-ALL cells to dasatinib. (A) Viability assay of p190 cells treated with vehicle (DMSO) or dasatinib (5 nM) and

the indicated inhibitors (silvestrol, 10 nM; hippuristanol, 100 nM; MLN0128, 100 nM). (B-E) Viability assays of human Ph+ (SUP-B15, BV173) and Ph-like (TVA1) B-ALL cell lines treated with different concentrations of dasatinib alone or with the indicated inhibitors of mTOR (MLN0128, 100 nM) or eIF4A (silvestrol, hippuristanol). Significance was calculated using two-way ANOVA (* $p < 0.5$, ** $p < 0.01$, and *** $p < 0.001$. Panel A: n = 3 for vehicle and MLN0128, 3 for Silvestrol and hippuristanol. Panel B: n = 5. Panel C, D and E: n = 3.).

Figure S4: Inhibition of mTORC1 or eIF4F suppresses colony formation by BCR-ABL-expressing mouse bone marrow cells.

Colony forming assay of BCR-ABL transformed mouse bone marrow cells (n=3) in Methocult media with increasing concentrations of Dasatinib, SBI-756, RapaLink compounds (E1035 and M1071), and MLN0128 (n = 1 for the 30 nM condition). Mean colony counts after 9 days were normalized to DMSO control. Significance was calculated using unpaired t-test (* $p < 0.05$, ** $p < 0.01$, *** $p < 0.001$, **** $p < 0.0001$, n = 3 except for the 30 nM MLN0128 condition, n = 1).

Supplemental Figure 1

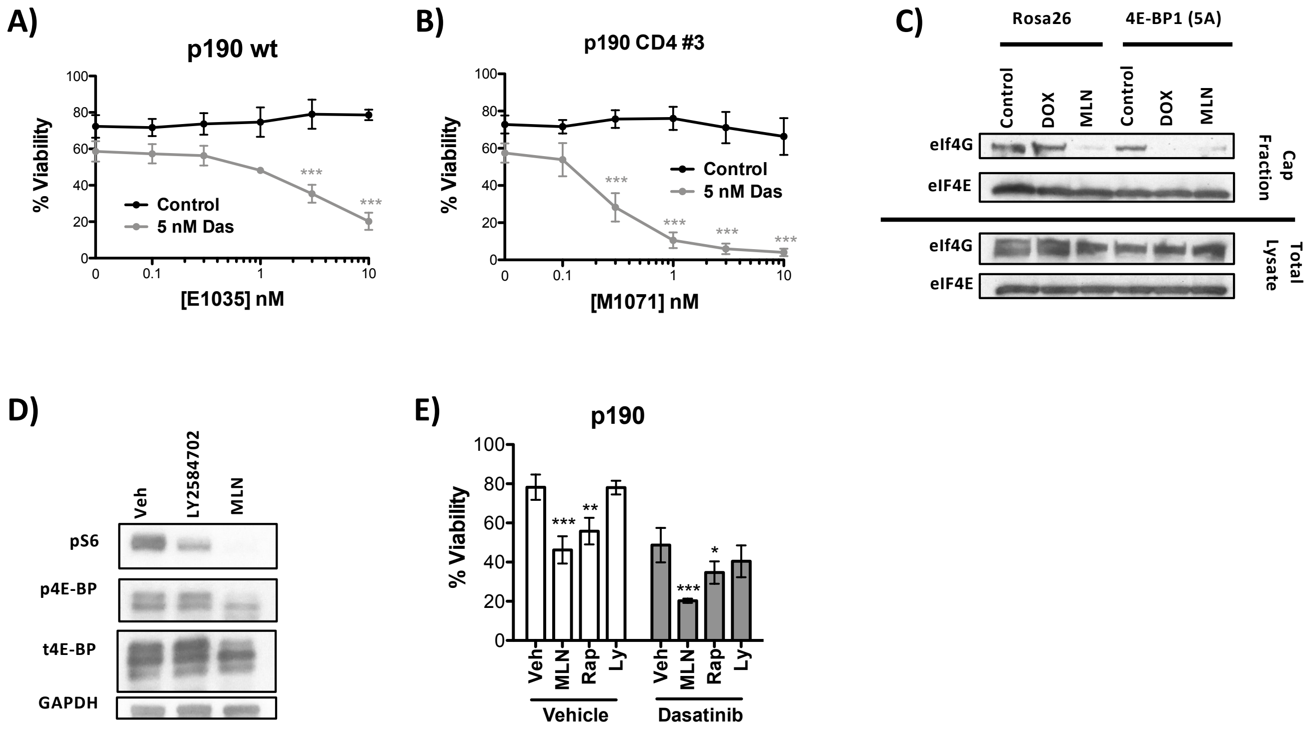

Supplemental Figure 2

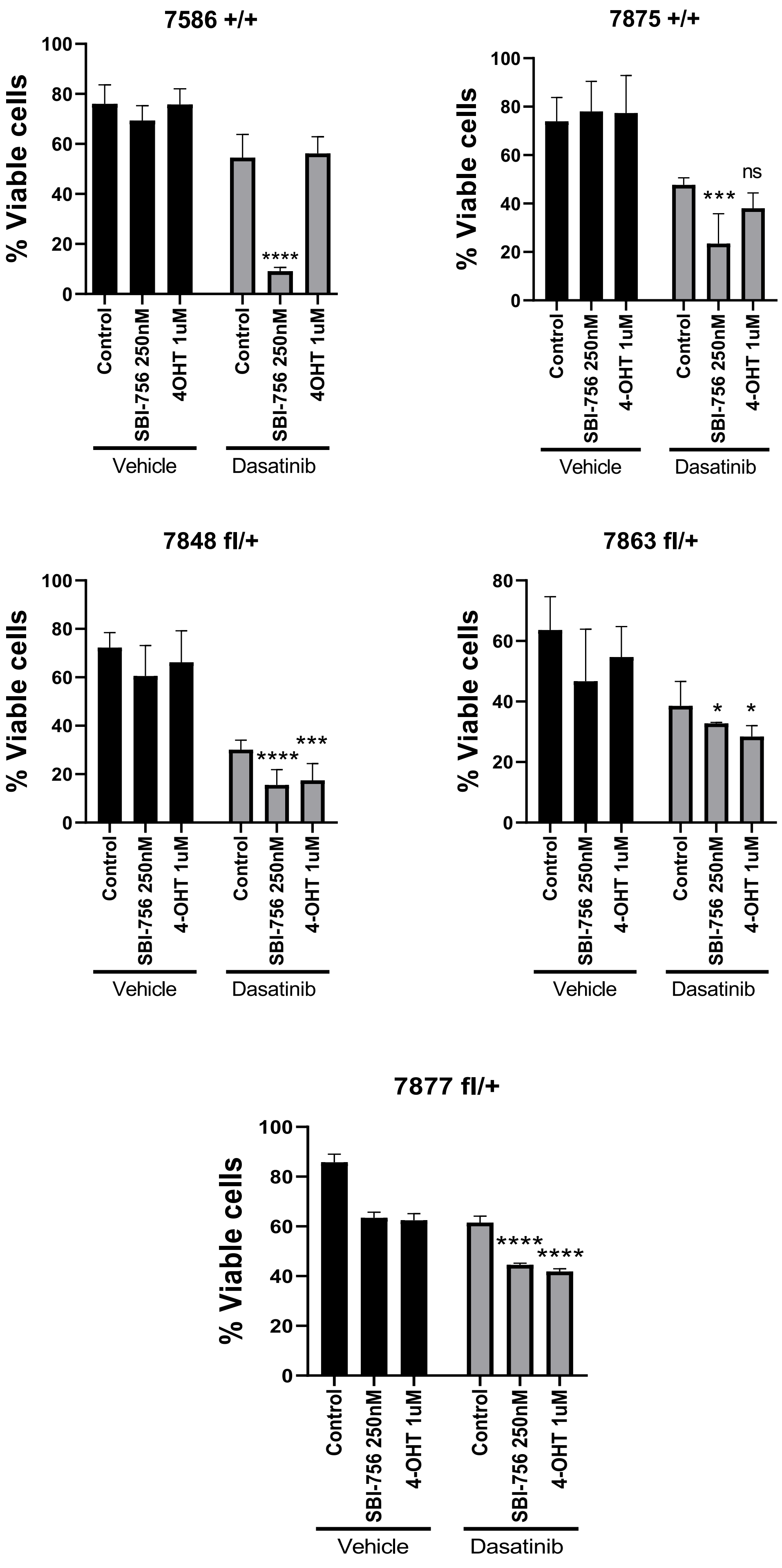

Supplemental Figure 3

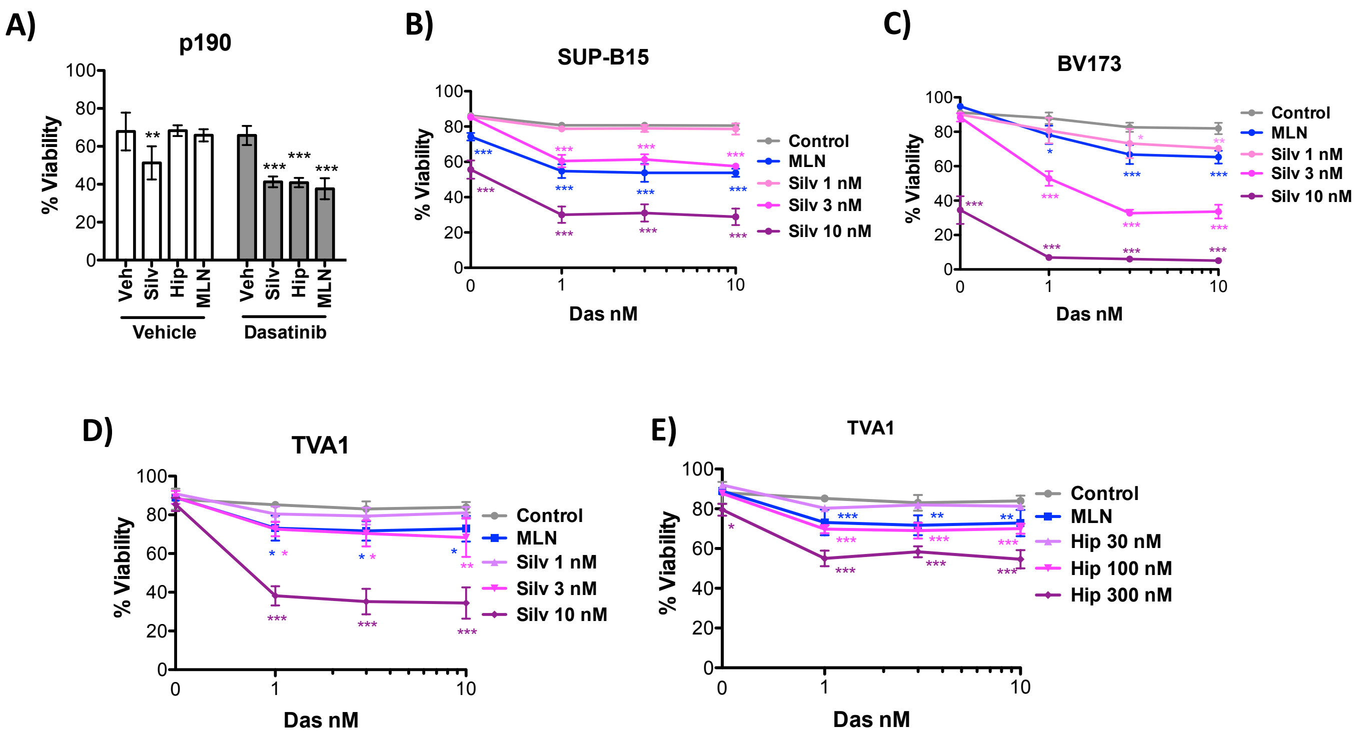

Supplemental Figure 4

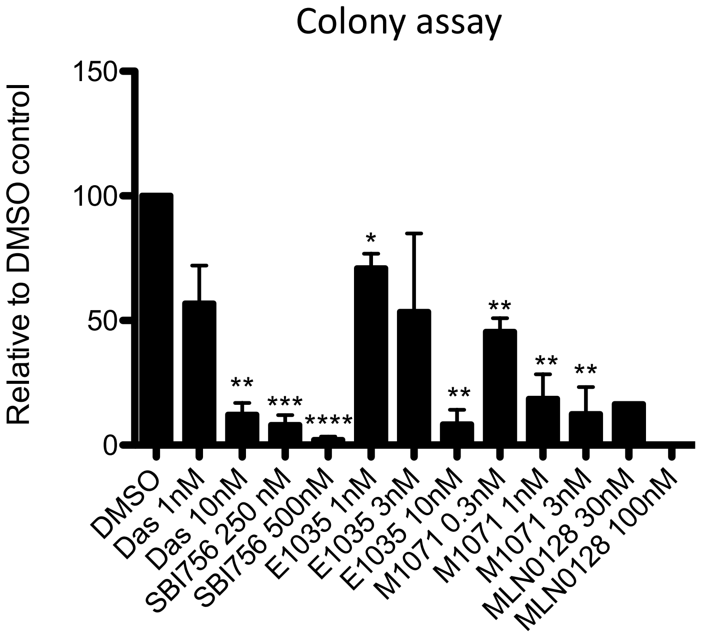

Original uncropped Western blot images
Vo, Herzog et al. (Scientific Reports)

- Each slide contains the following information:
  - On the left side, a reproduction of the figure panel from the main or supplementary figure
  - On the right side, the original digital image of the blot strips using chemoluminescent detection. The image is collected with white on a black background and inverted into black on white for the figures.
  - For all but one experiment (Figure S1C), we also include a photo of the blot strips with visible light.

Figure 1B

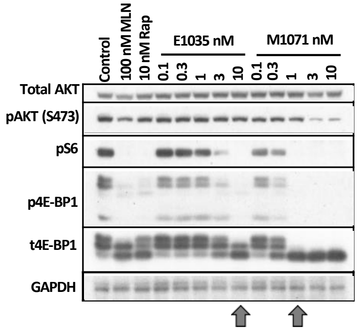
Control
100 nM MLN
10 nM Rap
E1035 nM
M1071 nM
0.1
0.3
1
3
10
0.1
0.3
1
3
10
Total AKT
pAKT (S473)
pS6
p4E-BP1
t4E-BP1
GAPDH

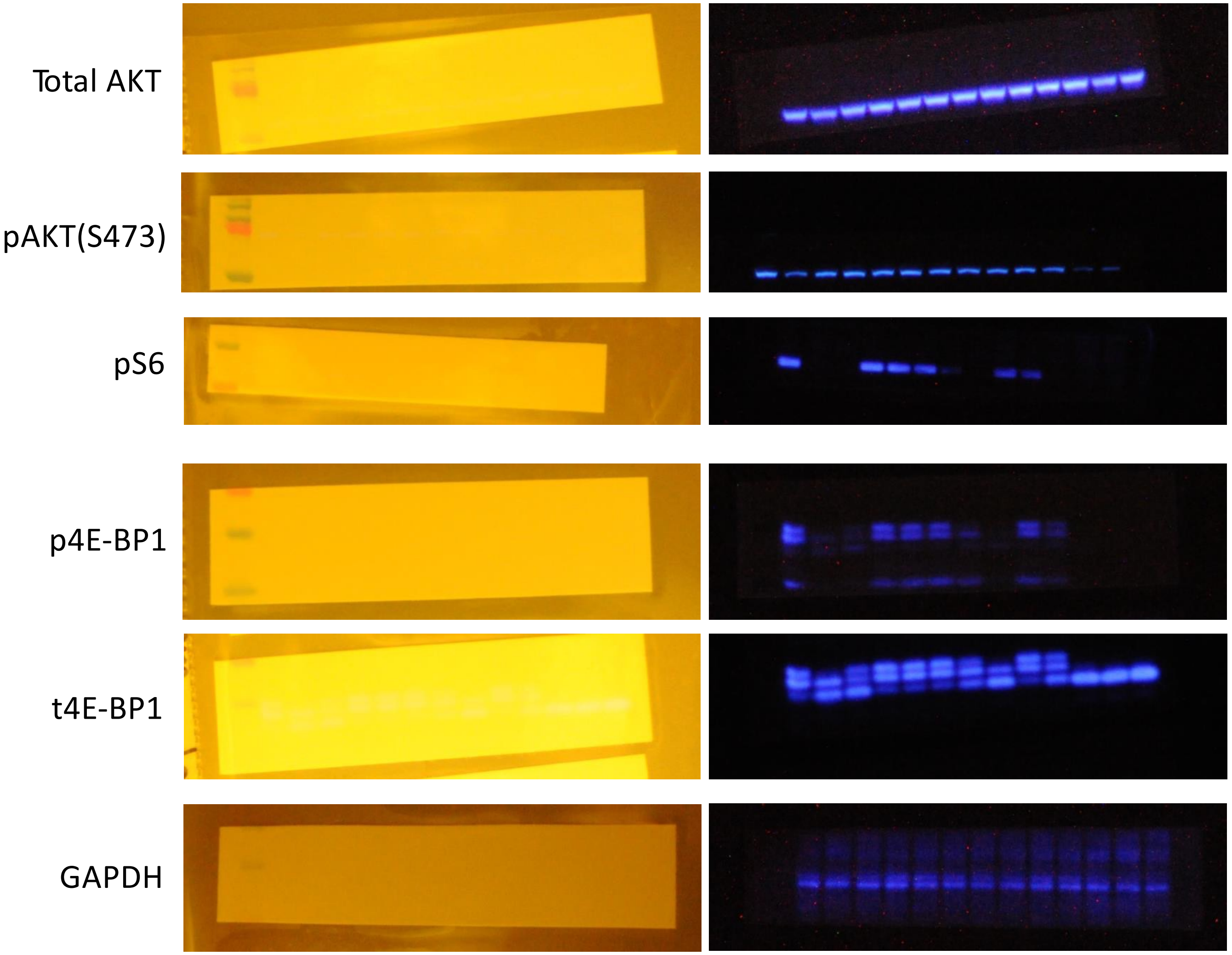
Total AKT
pAKT(S473)
pS6
p4E-BP1
t4E-BP1
GAPDH

Figure 1D

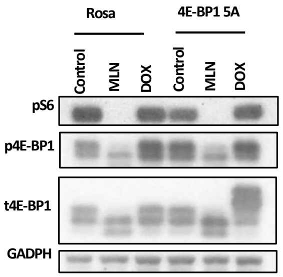
Rosa
4E-BP1 5A
Control
MLN
DOX
Control
MLN
DOX
pS6
p4E-BP1
t4E-BP1
GADPH

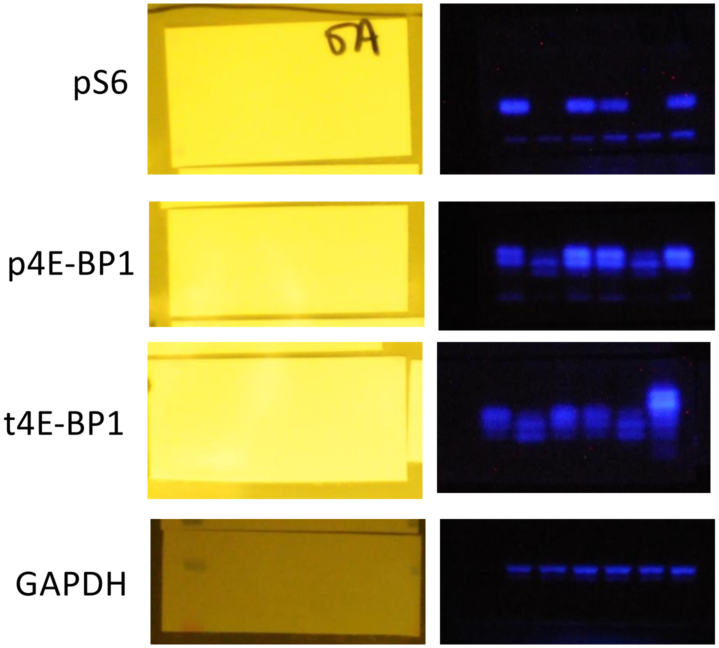
pS6
p4E-BP1
t4E-BP1
GAPDH

Figure 2A

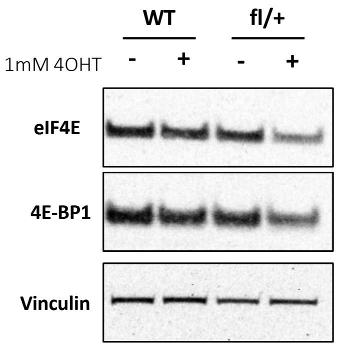

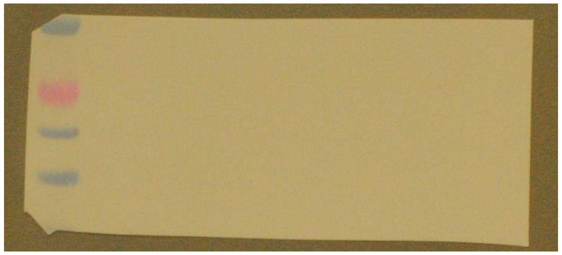

eIF4E

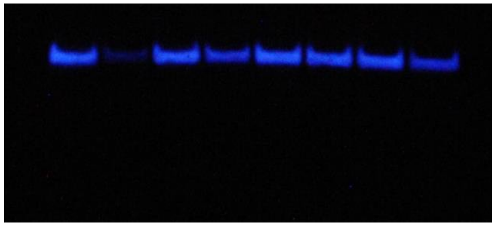

4E-BP1

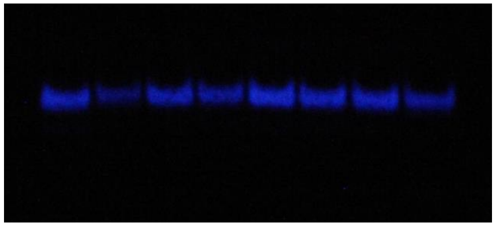

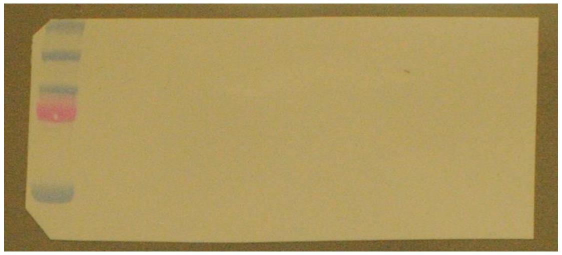

Vinculin

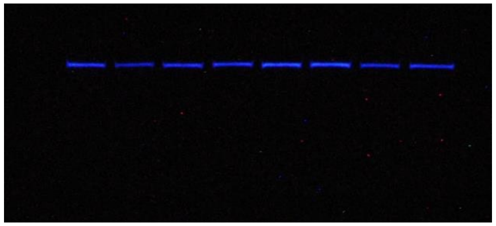

Figure 3C

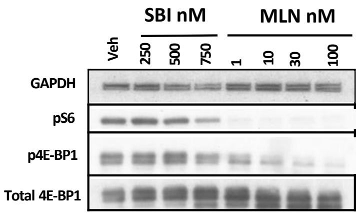
SBI nM
MLN nM
Veh
250
500
750
1
10
30
100
GAPDH
pS6
p4E-BP1
Total 4E-BP1

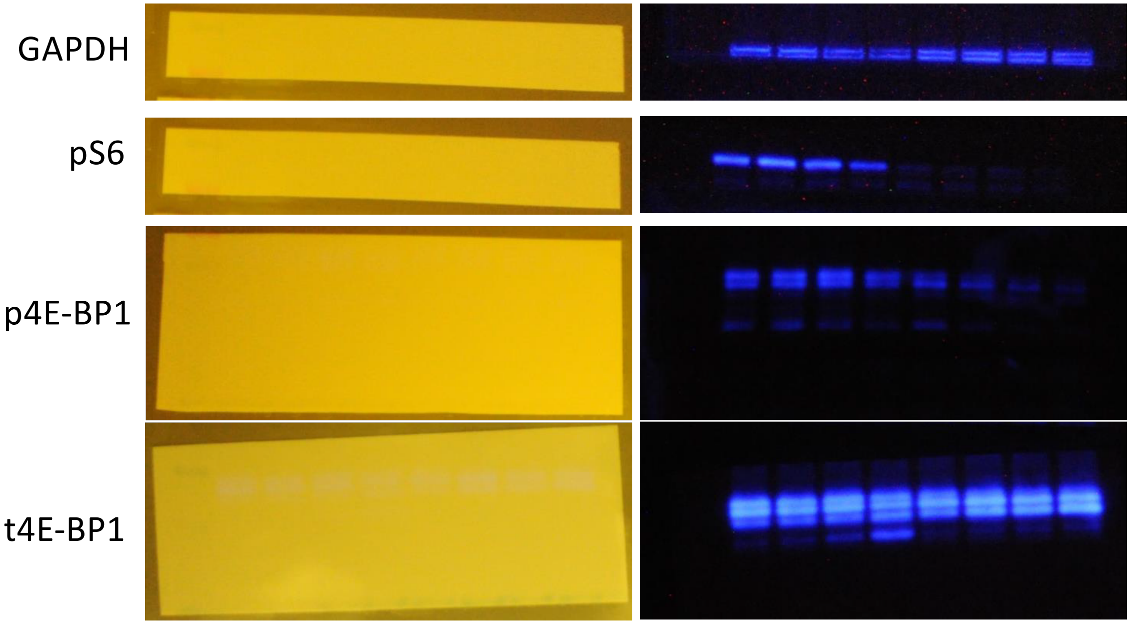
GAPDH
pS6
p4E-BP1
t4E-BP1

SupFig 1C

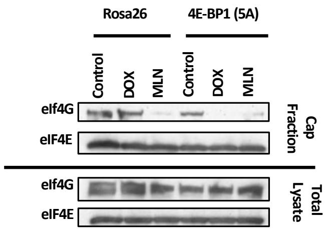

Cap eIF4G

Cap eIF4E

Total eIF4G

Total eIF4E

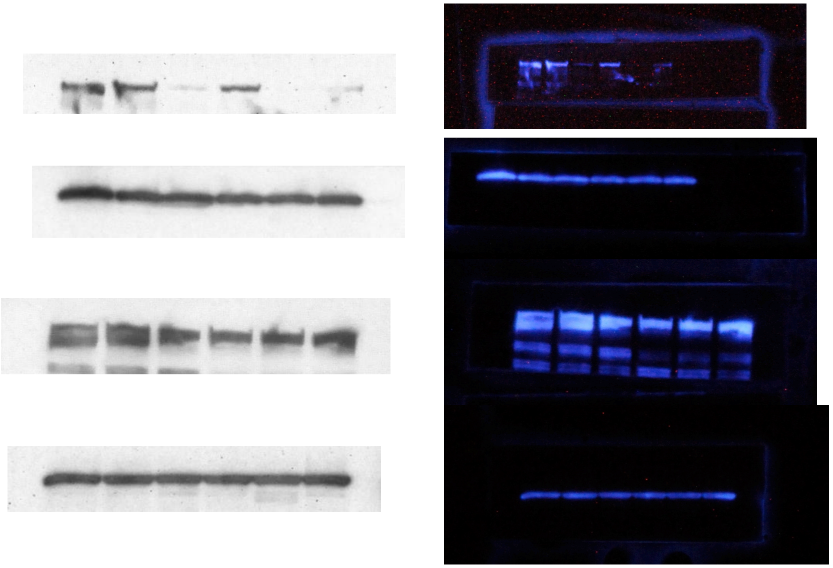

Photos of the blot strips with visible light were not saved for this experiment.

SupFig 1D

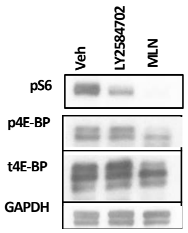
Veh
LY2584702
MLN
pS6
p4E-BP
t4E-BP
GAPDH

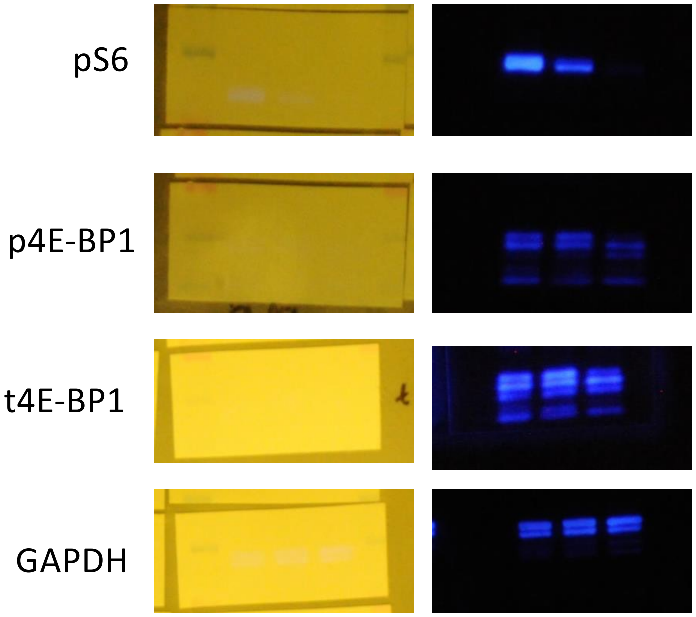
pS6
p4E-BP1
t4E-BP1
GAPDH
